# Patterns in Prescribing and Predictors of SGLT2 Inhibitor Administration in Patients with Heart Failure and Acute Myocardial Infarction: A Real-World Retrospective Cohort Study

**DOI:** 10.3390/jcm15031056

**Published:** 2026-01-28

**Authors:** Ioana Maria Suciu, Teodora Mateoc-Sîrb, Constantin Tudor Luca, Bogdan Timar, Dan Gaiță

**Affiliations:** 1 Doctoral School, “Victor Babeș” University of Medicine and Pharmacy, Eftimie Murgu Sq. No. 2, 300041 Timișoara, Romania; ioana-maria.suciu@umft.ro; 2 Institute of Cardiovascular Diseases Timisoara, Gh. Adam, 13A, 300310 Timișoara, Romania; 3 Research Center of the Institute of Cardiovascular Diseases Timișoara, Gh. Adam, 13A, 300310 Timișoara, Romania; 4 Cardiology Department, “Victor Babeș” University of Medicine and Pharmacy, Eftimie Murgu Sq. No. 2, 300041 Timișoara, Romania; 5 Department of Diabetes, Nutrition and Metabolic Diseases Clinic, “Pius Brînzeu” Emergency Clinical County University Hospital, 300723 Timișoara, Romania; 6 Department of Second Internal Medicine—Diabetes, Nutrition, Metabolic Diseases and Systemic Rheumatology, “Victor Babeș” University of Medicine and Pharmacy, Eftimie Murgu Sq. No. 2, 300041 Timișoara, Romania

**Keywords:** SGLT2 inhibitors, acute myocardial infarction, prescription patterns

## Abstract

**Background/Objectives:** Sodium-glucose cotransporter 2 (SGLT2) inhibitors provide well-established cardiovascular and renal benefits in heart failure (HF), type 2 diabetes (T2DM), and chronic kidney disease (CKD). Although emerging trials suggest potential value after acute myocardial infarction (AMI), SGLT2 inhibitors currently have no formal indication for AMI, and real-world prescribing patterns in this setting remain uncharacterized. This study aimed to evaluate in-hospital and post-discharge prescribing patterns and clinical predictors of SGLT2 inhibitor initiation among AMI patients eligible for therapy based on guideline-supported indications. **Methods:** We conducted a retrospective cohort study including 244 consecutive AMI patients hospitalized between January 2023 and July 2024. A total of 180 (73.7%) met guideline-based eligibility criteria for SGLT2 inhibitors. Four multivariable logistic regression models were developed to identify independent predictors of SGLT2 inhibitor prescription. **Results:** A total of 117 patients (65%) received SGLT2 inhibitors and 63 (35%) remained untreated. Receivers were more frequently male (81% vs. 65%) and exhibited lower left ventricular ejection fraction (LVEF) (38.2 ± 6.7% vs. 42.4 ± 8.3%), larger ventricular volumes, and higher Killip class at presentation. HF patients with preserved ejection fraction (HFpEF) were markedly undertreated (25.9%) compared with mid-range (HFmrEF) (69.8%) or reduced (HFrEF) (73.7%). Across all models, HFpEF was a strong negative predictor of prescribing (OR 0.071–0.081, *p* < 0.001), while male sex and markers of clinical severity were associated with higher likelihood of initiation. Many untreated patients had T2DM or CKD despite guideline-based eligibility. No serious adverse events attributable to SGLT2 inhibitors were reported. **Conclusions:** In this real-world AMI cohort, SGLT2 inhibitors were prescribed primarily in relation to established indications for HF, T2DM, and CKD, yet their use remained highly variable in the absence of a dedicated recommendation for AMI. Significant therapeutic gaps were observed in HFpEF and high-risk cardiometabolic profiles, underscoring the need for clearer guidance and standardized pathways to support consistent initiation in eligible patients after MI.

## 1. Introduction

During hospitalization for acute myocardial infarction (AMI), within a short and intensive care window, clinicians must rapidly stabilize the patient and, beyond ensuring revascularization and optimizing antithrombotic therapy, modern secondary prevention increasingly incorporates adjusting multiple chronic treatments, such as long-term cardiometabolic treatments that improve survival and reduce rehospitalization [1,2]. In this clinical context, decisions regarding the initiation of additional therapies must balance guideline recommendations, hemodynamic stability, renal function, and patient-related factors.

In patients with established atherosclerotic cardiovascular disease, sodium-glucose cotransporter 2 (SGLT2) inhibitors represent guideline-supported therapy when traditional indications are present: type 2 diabetes mellitus (T2DM), chronic kidney disease (CKD), and heart failure (HF), independent of glycemic control [3]. Their cardiovascular and renal benefits are well established across multiple HF large-scale trials, but emerging data emphasize additional benefits if administered in patients with AMI [4,5,6,7,8].

However, the optimal timing for initiating SGLT2 inhibitors after AMI remains an area of ongoing investigation, and current practice is largely guided by established indications and clinical stability [9,10,11,12].

Recent dedicated post-MI trials, including DAPA-MI and EMPACT-MI, have stimulated growing interest in early initiation strategies [4,5]. These studies suggest potential benefits for cardiometabolic profiles, HF-related outcomes, and overall recovery, although SGLT2 inhibitors are not yet specifically indicated for the treatment of AMI itself.

Consequently, clinicians may consider introducing SGLT2 inhibitors in eligible patients, particularly those who already meet guideline-based criteria, when clinical stability is reached [13,14,15].

Understanding how often such early in-hospital initiation is feasible in real-world practice, and in what types of patient clinicians choose this strategy, is important for several reasons. First, it provides insights into contemporary implementation of evidence-based therapies. Second, it helps identify patient profiles in which early initiation is perceived as clinically appropriate and safe. Finally, it may highlight opportunities to optimize the integration of SGLT2 inhibitors into holistic post-MI management, without implying underuse or deviation from guidelines [16,17,18,19,20,21].

Because AMI patients commonly exhibit diabetes, subclinical or overt heart failure, impaired renal function, and metabolic risk, they constitute a population expected to benefit substantially from SGLT2 inhibitors [22,23,24]. Despite emerging data supporting their role in the setting of AMI, randomized controlled trials (RCTs) and registry data suggest poor real-world uptake, particularly in obese patients or those with preserved LVEF, a population which may benefit most from this therapy [25].

Beyond glucose lowering, SGLT2 inhibitors exert pleiotropic effects including anti-inflammatory, metabolic, and myocardial protective mechanisms, which may be particularly relevant in the ACS setting [26].

## 2. Materials and Methods

### 2.1. Study Design and Population

We conducted a retrospective, observational cohort study including 244 consecutive adult patients (aged ≥ 18 years) admitted with AMI between January 2023 and July 2024 at a tertiary cardiovascular center (Institute of Cardiovascular Diseases, Timisoara, Romania). AMI was defined according to the Fourth Universal Definition of Myocardial Infarction. Eligibility for SGLT2 inhibitor therapy was assessed based on contemporary guideline indications: HF, T2DM, or CKD. Of the total cohort, 180 patients (73.7%) met at least one eligibility criterion and were included in the cohort.

### 2.2. Definition of Exposure

Patients were categorized into two groups: SGLT2 inhibitor receivers, defined as those who initiated treatment either during the index hospitalization or after discharge during follow-up, and non-receivers, defined as eligible patients who did not receive SGLT2 inhibitors during the study period. This study evaluates prescribing patterns and timing of initiation in routine practice and does not aim to define clinically validated “early” versus “delayed” treatment strategies based on outcome-driven follow-up benchmarks. Both dapagliflozin and empagliflozin were available for prescription according to physician judgment.

### 2.3. Data Collection

Electronic medical records were reviewed to extract demographic data, cardiovascular risk factors, comorbidities, admission clinical parameters, laboratory tests, echocardiographic measurements, and pharmacological treatments. Echocardiographic parameters were obtained by accredited cardiologists using standardized acquisition protocols. HF phenotype was classified using the left ventricular (LV) ejection fraction (EF) as reduced < 40% (HFrEF), mid-range 40–49% (HFmrEF), or ≥50% preserved (HFpEF) according to ESC 2021 criteria. CKD was defined as eGFR ≤ 60 mL/min/1.73 m^2^. Echocardiography was performed within 1 h after admission (baseline) and repeated at follow-up using Vivid S5, GE Healthcare, Chicago, IL, USA.

### 2.4. Outcomes

The primary objective was to characterize prescribing patterns of SGLT2 inhibitors in AMI. Secondary objectives included identifying independent predictors of SGLT2 inhibitor use and describing clinical factors associated with undertreatment.

### 2.5. Statistical Analysis

Continuous variables were compared using Student’s *t*-test or the Mann–Whitney U test, as appropriate. Categorical variables were compared with the chi-square or Fisher’s exact test. For variables with non-normal distribution, medians and interquartile ranges were reported.

To identify independent predictors of SGLT2 inhibitor prescription, we constructed four multivariable logistic regression models incorporating demographic, clinical, echocardiographic, and therapeutic variables. Odds ratios (ORs) with 95% confidence intervals (CIs) were reported. A two-sided *p*-value < 0.05 was considered statistically significant. Analyses were performed using R statistical software (version 4.5.1). A hierarchical modeling strategy was used, building four concept-driven regression models. Model 1 (AMI severity model) was adjusted for infarction characteristics (STEMI, anterior MI), Killip class, and length of stay. Model 2 (cardiometabolic model) included age, sex, diabetes, hypertension, obesity, smoking, eGFR, NLR, and prior CAD. Model 3 (heart failure phenotype and guideline-directed therapy model) included HF phenotype (HFrEF, HFmrEF, HFpEF), Killip class, and use of ACEi/ARB/ARNi, beta-blockers, and MRA. Model 4 (fully adjusted model) combined all clinically relevant predictors from the previous models (age, sex, diabetes, obesity, eGFR, HF phenotype, Killip class, STEMI, and guideline-directed HF therapies). Model 4 was considered the primary analysis, while Models 1–3 were treated as secondary exploratory models.

Model diagnostics were systematically assessed for all multivariable logistic regression models. Multicollinearity was evaluated using variance inflation factors (VIFs); for categorical predictors with more than two levels, generalized VIF adjusted for degrees of freedom (GVIF^(1/(2×df))^) was used. Model calibration was assessed using the Hosmer–Lemeshow goodness-of-fit test, and discrimination was evaluated using the area under the receiver operating characteristic curve (AUC). Diagnostic results for all models are reported in the Supplementary Materials (Table S1).

### 2.6. Ethics

This study complied with the Declaration of Helsinki and was approved by the institutional ethics committee (Nr. 14/10.01.2024, 10 January 2024). Given the retrospective design, informed consent was obtained at admission in the medical chart.

## 3. Results

### 3.1. Summary of the Included Participants

**Figure 1:**
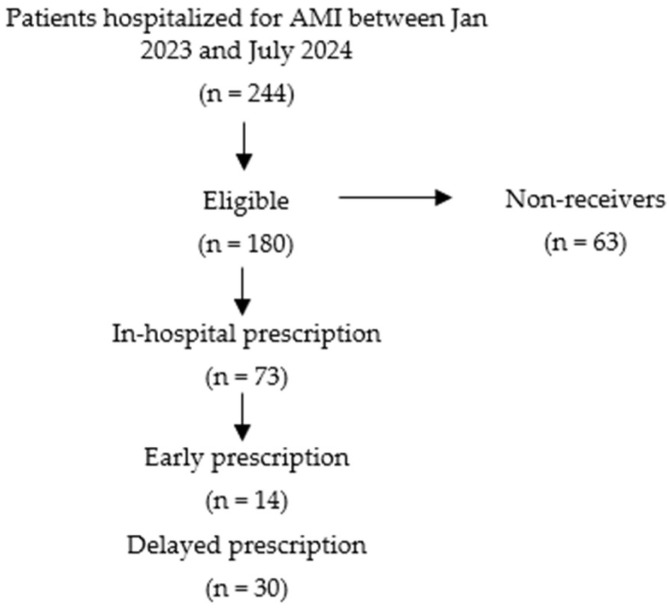
Selection process.

Among 244 patients hospitalized for AMI (Figure 1), 180 (73.7%) met at least one of the guideline-based eligibility criteria for SGLT2 inhibitors. Of these, 117 (65%) received an SGLT2 inhibitor during hospitalization or follow-up, while 63 (35%) did not. Baseline characteristics are summarized in Table 1.

#### 3.1.1. Demographics and Comorbidities

Patients receiving SGLT2 inhibitors were slightly younger (60.85 ± 9.82 vs. 63.67 ± 11.58 years, *p* = 0.046) and more frequently male (81% vs. 65%, *p* = 0.016). Obesity and a higher grade of hypertension were more common among untreated patients (44% vs. 29%, *p* = 0.038; and 41% vs. 26%, *p* = 0.031), indicating a moderate baseline imbalance.

A notable difference was observed for CKD (eGFR ≤ 60 mL/min/1.73 m^2^) which was significantly more prevalent among untreated patients (30.1% vs. 12.8%, *p* = 0.004), indicating underutilization in a population with strong guideline recommendations for therapy. Other major comorbidities, including T2DM (51% vs. 44%, *p* = 0.40) and prior MI, were broadly similar between groups.

#### 3.1.2. Admission Characteristics

Hemodynamic parameters at admission were comparable between groups. STEMI was the predominant presentation in both groups (88% vs. 89%, *p* = 0.86). However, anterior wall MI was more common in the SGLT2 inhibitor group (48% vs. 32%, *p* = 0.03), while inferior wall MI was numerically more frequent among non-receivers (51% vs. 37%, *p* = 0.06). The number of damaged vessels and the number of stents implanted at the index procedure were similar.

Killip class ≥ II was significantly more frequent among treated patients (31 vs. 8; *p* = 0.03), suggesting that SGLT2 inhibitors were more often prescribed in patients with more symptomatic HF at presentation, which introduces a potential confounding by disease severity. Length of hospitalization showed no difference (median 5 [3–7] vs. 4 [3–7] days, *p* = 0.73).

#### 3.1.3. Laboratory Parameters

Hemoglobin levels showed a borderline difference (14.62 ± 1.42 vs. 14.11 ± 1.60 g/dL, *p* = 0.053), while inflammatory markers (NLR, PLR, uric acid), electrolytes, glycemia, and eGFR were comparable. Troponin levels were numerically higher in the treated group but without statistical significance (*p* = 0.12).

#### 3.1.4. Medications During Hospitalization

Use of ACEi/ARB/ARNI (73% vs. 68%, *p* = 0.5), β-blockers (78% vs. 78%, *p* = 0.9), MRAs (87% vs. 76%, *p* = 0.059), and loop diuretics (83% vs. 78%, *p* = 0.4) did not differ significantly.

Antiplatelet therapy showed a trend toward more frequent ticagrelor use in the SGLT2 inhibitor group (56% vs. 41%) and reciprocal clopidogrel use in untreated patients (59% vs. 44%; *p* = 0.052). This pattern suggests that SGLT2 inhibitors were more often prescribed in patients managed with more contemporary ACS regimens.

#### 3.1.5. Echocardiography

Left ventricular systolic function was more impaired among patients receiving SGLT2 inhibitors, evidenced by lower LVEF (38.21 ± 6.71% vs. 42.43 ± 8.34%, *p* = 0.001) and larger LVEDV (119.64 ± 45.59 vs. 106.81 ± 38.42 mL, *p* = 0.008). LVM and LVEDD were similar.

Although LV end-diastolic volumes were larger in the treated group, this finding should be interpreted cautiously, as this group also included a higher proportion of male patients. Accordingly, LV size parameters were not emphasized as independent predictors of SGLT2 inhibitor prescription.

HF phenotype distribution differed markedly. HFpEF was more common in the untreated group (31.7% vs. 6%), whereas HFrEF was more common among treated patients (50% vs. 33%). These imbalances underscore the need for careful adjustment, as HF phenotype and LVEF represent major confounders and appropriately enter the multivariable models.

#### 3.1.6. Eligibility and Prescription Flow

Among the 180 eligible patients, 40.6% initiated SGLT2 inhibitors during the index hospitalization (at discharge). Additional treatment initiation occurred after discharge during follow-up and was described as post-discharge initiation at the first outpatient reassessment (7.7%; median 50 days, IQR 44–61) and later outpatient initiation (16.6%; median 386 days, IQR 322–516). These time windows reflect observed clinical practice rather than predefined, guideline-endorsed timing benchmarks. Delayed initiation was most commonly attributed to transient clinical contraindications in 28 patients, including hemodynamic compromise requiring inotropes or associated with cardiac arrest or significant hypotension (*n* = 9, 32.1%), severe infection (*n* = 11, 39.28%), AKI (*n* = 3, 10.71%), DKA (*n* = 3, 10.71%), or UTIs (*n* = 2, 7.14%). These findings suggest that deferral was frequently clinically justified.

### 3.2. SGLT2 Inhibitor Use According to HF Phenotype

Across the cohort, the overall SGLT2 inhibitor initiation rate was 65%. They were more frequently administered in addition to all three foundational GDMT components: ACEi/ARNi (77.7% vs. 68.3%, *p* = 0.16), BB (77.7% vs. 76.2%, *p* = 0.8), and MRA (87.2% vs. 77.7%, *p* = 0.1), although none of these differences reached statistical significance. This pattern suggests that SGLT2 inhibitors were more often prescribed in patients already managed within a more aggressive HF treatment strategy.

#### 3.2.1. HFrEF

Among the 80 patients with HFrEF, the use of SGLT2 inhibitors (73.7%) was associated with a more intensive HF pharmacological profile. ACEi/ARNi therapy was significantly more frequent among SGLT2 inhibitot recipients (*p* = 0.026), indicating a higher likelihood of receiving optimized foundational therapy.

##### Evolution of HF Phenotype in HFrEF

During follow-up, patients receiving SGLT2 inhibitors tended to remain in the HFrEF category more commonly than untreated patients (30 vs. 6 patients; *p* = 0.078). Transitions to HFmrEF occurred at similar rates (*p* = 0.67), whereas transition to HFpEF was more frequent among non-recipients (*p* = 0.033). Although the sample size limits definitive interpretation, these findings do not suggest a more favorable ventricular remodeling trajectory in patients treated with SGLT2 inhibitors (Table 2).

#### 3.2.2. HFmrEF and HFpEF

Among the 100 patients classified as HFmrEF or HFpEF, prescription of SGLT2 inhibitors differed substantially between phenotypes. Uptake was considerably higher in HFmrEF (69.9%), whereas only 25.9% of HFpEF patients received an SGLT2 inhibitor. This gradient aligns with differences in prescribing behavior and clinician familiarity across HF phenotypes.

##### Evolution of HF Phenotype in HFmrEF and HFpEF

Transitions across HF phenotypes were broadly similar between patients with and without SGLT2 inhibitors. In HFmrEF, no significant differences were observed in transitions to HFrEF, persistence in HFmrEF, or progression to HFpEF. In HFpEF, transitions toward HFmrEF or HFrEF were likewise comparable between groups.

Overall, SGLT2 inhibitor therapy was not associated with statistically significant differences in functional HF phenotype transitions, although directional changes tended to be neutral (Table 3).

#### 3.2.3. GDMT Distribution by Heart Failure Phenotype

Table 4 summarizes the distribution of guideline-directed medical therapy (GDMT) according to HF phenotypes. To allow the assessment of treatment optimization, guideline-directed medical therapy (GDMT) was categorized into foundational GDMT (ACEI/ARB/ARNI + β-blocker + MRA) and extended GDMT (foundational GDMT + SGLT2 inhibitor).

GDMT prescription patterns varied markedly across HF phenotypes. Foundational GDMT (three pillars) was achieved in 13.8% of the overall HF population, while extended GDMT (four pillars) was achieved in 37.8%.

In HFrEF, extended GDMT was four times more common than foundational GDMT (45% vs. 11.3%).

In HFmrEF + HFpEF, extended GDMT (32%) was also more frequent than foundational GDMT (16%), although markedly lower than in HFrEF. HFpEF remained the least treated phenotype, reflecting persistent therapeutic inertia.

These findings highlight that treatment optimization was most consistently achieved in HFrEF, where guideline recommendations are strongest. In contrast, HFmrEF and especially HFpEF patients remained substantially undertreated, despite Class II guideline recommendations and robust evidence for SGLT2 inhibitor benefit in this phenotype.

Although all HF patients had universal guideline eligibility for SGLT2 inhibitors, prescribing followed a strong phenotype-dependent pattern: high in HfrEF (73.7%), moderate in HfmrEF (69.9%), and minimal in HFpEF (25.92%). Extended GDMT was more common than foundational GDMT, but almost exclusively in HFrEF and HFmrEF, indicating selective intensification of therapy rather than uniform application across HF phenotypes.

SGLT2 inhibitors were frequently initiated during the index hospitalization or after discharge in eligible patients. However, given the observational design, no inference regarding efficacy or outcomes can be made. Initiation remained highly phenotype-dependent, with lower use in HFpEF despite guideline-based eligibility.

### 3.3. Prescription Patterns According to T2DM or CKD

Most patients with HF and concomitant T2DM or CKD received therapy during hospitalization or follow-up. However, a subset of patients with these comorbidities did not receive SGLT2 inhibitors, suggesting that although prescribing practices were generally aligned with guideline recommendations, opportunities for further optimization remained.

Among untreated patients with HFrEF (*n* = 21), several presented with metabolic or renal profiles that would typically reinforce the indication for SGLT2 inhibitors: eight had T2DM, seven had CKD, and five exhibited the combined pattern of obesity with either T2DM or CKD. In the group with HF > 40% (*n* = 42), twenty-four untreated patients had T2DM, twelve had CKD, and seventeen had obesity in addition to T2DM or CKD.

Overall, these findings indicate that SGLT2 inhibitor therapy was broadly implemented in patients with cardiometabolic comorbidities, yet a non-negligible proportion of high-risk individuals, particularly those with overlapping obesity and metabolic or renal disease, remained untreated. This pattern suggests that clinical practice is largely consistent with guideline-directed care, while also highlighting areas where SGLT2 inhibitor use might be further expanded to maximize benefit in high-risk profiles.

### 3.4. Predictors of SGLT2 Inhibitor Use

Four multivariable logistic regression models were constructed to explore independent predictors of SGLT2 inhibitor prescription.

#### 3.4.1. Model 1 (MI Severity)

Killip class was the only independent predictor. Each increase in Killip class was associated with a higher probability of receiving SGLT2 inhibitors (OR = 2.05, 95% CI 1.10–4.29, *p* = 0.036). Male sex showed a borderline association (OR 2.08, 95% CI 0.98–4.44, *p* = 0.056). Age, STEMI presentation, anterior MI, number of damaged vessels, and length of hospitalization were not predictive (all *p* > 0.15) (Table 5).

#### 3.4.2. Model 2 (Comorbidities and Risk Factors)

Male sex and obesity were the main determinants. Male patients had nearly twice the odds of receiving SGLT2 inhibitors (OR = 2.19, 95% CI 1.05–4.57, *p* = 0.039), whereas obesity was inversely associated with treatment (OR = 0.43, 95% CI 0.21–0.87, *p* = 0.020). Other comorbidities (diabetes, hypertension, old CAD) and biomarkers (NLR, eGFR) were not significant, suggesting that treatment decisions were not primarily driven by traditional cardiometabolic risk factors, but rather by phenotype and clinical perception (Table 6).

#### 3.4.3. Model 3 (Medication and HF Phenotype)

When HF phenotype and background HF therapy were included, HFpEF emerged as a strong independent negative predictor of SGLT2i use (OR 0.071, 95% CI 0.015–0.268; *p* = 0.00028). Age (OR 0.966, 95% CI 0.933–0.999; *p* = 0.0486) and male sex (OR 2.64, 95% CI 1.21–5.85; *p* = 0.0155) also remained significant. HFmrEF had no independent effect (OR 0.92, 95% CI 0.43–1.96; *p* = 0.82). Killip class and ACEi/ARB/ARNi use showed non-significant trends (OR 1.73 and 2.06, respectively) (Table 7).

#### 3.4.4. Model 4 (Adjusted Clinical Model)

In the fully-adjusted model combining key variables from Models 1–3, HFpEF remained the strongest negative predictor of SGLT2 inhibitor prescription (OR 0.081, 95% CI 0.016–0.317; *p* = 0.00075), while male sex persisted as a positive predictor (OR 2.58, 95% CI 1.14–5.93; *p* = 0.023). Killip class showed a borderline association (OR 1.71, 95% CI 0.93–3.46; *p* = 0.106), as did concomitant ACEi/ARB/ARNi use (OR 2.17, 95% CI 0.97–4.89; *p* = 0.059). Obesity was no longer statistically significant (OR 0.61, 95% CI 0.29–1.32; *p* = 0.207). These models consistently indicate that HF phenotype (especially HFpEF), sex, and markers of clinical severity are the main drivers of SGLT2 inhibitor prescribing, rather than traditional risk factors alone (Table 8).

### 3.5. Adverse Events and Mortality

Ten patients discontinued SGLT2 inhibitor therapy during follow-up, most commonly due to symptomatic hypotension (*n* = 6) or recurrent urinary tract infections (*n* = 4). No serious adverse events, such as diabetic ketoacidosis or severe acute kidney injury, were observed. Mortality during follow-up was low with one death having occurred in the SGLT2 inhibitor group compared with three deaths in the non-SGLT2 inhibitor group.

## 4. Discussion

In this real-world cohort of patients with AMI, we observed an overall initiation rate of 65% of SGLT2 inhibitors, but with marked heterogeneity across HF phenotypes and cardiometabolic profiles, reflecting local prescribing practice within the study period.

Prescription was strongly driven by HF phenotype and comorbid T2DM or CKD. SGLT2 inhibitors were preferentially initiated in patients with more severe LV systolic dysfunction and higher Killip class, while HFpEF and obese patients with T2DM or CKD remained substantially undertreated. The inverse association between obesity and SGLT2 inhibitor prescription observed in our cohort aligns with prior observations describing a paradoxical undertreatment of obese patients, despite evidence that metabolic and weight-related mechanisms may enhance therapeutic benefit in this population [27,28].

Despite these selective patterns, initiation during hospitalization or during follow-up was not associated with major safety concerns in this cohort.

Although current guidelines do not provide a specific recommendation for routine SGLT2 inhibitor initiation in the acute MI setting, many patients of our cohort fulfilled strong, guideline-based indications for these drugs due to coexisting HF, T2DM, or CKD [1,3]. In this context, SGLT2 inhibitor prescription was frequently driven by the underlying cardiometabolic profile rather than the acute ischemic event itself.

Large randomized trials evaluating early SGLT2 inhibitor initiation after MI have suggested potential benefits for HF-related outcomes and cardiometabolic risk profiles, but have not yet translated into a formal guideline recommendation for systematic use in all post-MI patients [4,5,29,30]. As a result, in contemporary practice, SGLT2 inhibitors are usually prescribed based on established indications.

In our cohort, SGLT2 inhibitors were more often initiated in patients with more severe LV systolic dysfunction and higher Killip class, reflecting a confounding-by-indication pattern in which clinicians preferentially targeted individuals with manifest or incipient HF. Extended GDMT (four pillars) was more frequently achieved than foundational GDMT (three pillars), particularly in HFrEF, suggesting that once clinicians pursued comprehensive HF optimization, SGLT2 inhibitors were frequently integrated into the regimen [31,32,33,34]. However, this intensification remained highly phenotype-dependent and was far from uniform across the HF spectrum.

Although evidence supporting SGLT2 inhibitors has expanded across HF phenotypes, we observed substantially lower initiation rates in HFpEF [3]. Importantly, this association should be interpreted as a real-world prescribing pattern rather than a clinical contraindication, and it may reflect clinician hesitancy, therapeutic inertia, or uncertainty in the acute-care setting.

HFpEF patients were the least likely to receive SGLT2 inhibitors, and obese individuals with T2DM or CKD were disproportionately represented among untreated patients. This likely reflects a combination of therapeutic inertia, perceived uncertainty regarding the magnitude of benefit in HFpEF, and the absence of a dedicated acute MI indication, which together may limit physicians’ willingness to start SGLT2 inhibitors during the index hospitalization, even when eligibility is clear [35].

The index MI hospitalization offers a unique window for comprehensive initiation and up-titration of guideline-directed medical therapy. Deferring SGLT2 inhibitor initiation to outpatient follow-up risks therapeutic inertia, loss to follow-up, and delayed risk reduction, particularly in high-risk phenotypes [36,37,38]. Our data suggest that, in real-world practice, this opportunity is partially seized and SGLT2 inhibitors were often started early after AMI, but not consistently extended to all eligible subgroups.

At the same time, withholding therapy was frequently aligned with plausible safety concerns [39,40,41,42]. In keeping with previous trial observations, clinicians in our cohort commonly deferred SGLT2 inhibitor initiation in the presence of hemodynamic instability, acute kidney injury, diabetic ketoacidosis, or severe infection. This pattern suggests that non-prescription in certain cases was often clinically justified, rather than merely reflecting underuse. Importantly, no serious SGLT2 inhibitor-related adverse events such as diabetic ketoacidosis or severe AKI were observed, and discontinuation was mostly driven by hypotension or UTIs, supporting the overall safety of early post-MI use in appropriately selected patients.

Overall, our findings illustrate how, in the absence of a specific post-MI guideline indication, SGLT2 inhibitors are being selectively targeted to higher-risk HF phenotypes and cardiometabolic profiles, while substantial therapeutic gaps persist in HFpEF and in patients with T2DM or CKD who also have obesity. This selective adoption pattern is consistent with current guideline frameworks but highlights the potential for broader and more systematic implementation.

### 4.1. Study Limitations

This study has several limitations. First, its retrospective, single-center design limits causal inference and reduces generalizability. Treatment allocation was not randomized and depended on clinician judgment, resulting in confounding by indication: patients with more severe HF (lower LVEF, higher Killip class) were more likely to receive SGLT2 inhibitors. Although we used multivariable models to adjust for measured covariates, residual confounding from unmeasured factors, such as socioeconomic status, physician prescribing attitudes, volume status, or perceived clinical fragility, cannot be excluded.

Second, natriuretic peptide levels (BNP or NT-proBNP) were not systematically measured in all patients and were therefore not included in the analysis, which may have limited the characterization of heart failure severity, particularly in patients with preserved ejection fraction.

HF diagnosis and phenotype classification relied on routine clinical assessment and echocardiographic findings documented in the medical records, rather than on standardized diagnostic algorithms incorporating biomarkers or invasive hemodynamic testing. In particular, HFpEF diagnosis remains complex and cannot be fully captured by left ventricular ejection fraction alone.

Third, this study reflects real-world prescribing in a center without a standardized post-MI SGLT2 inhibitor protocol. As SGLT2 inhibitors are currently indicated for HF, T2DM, and CKD, but not specifically for acute MI, prescribing patterns may have been influenced by clinician familiarity, therapeutic inertia, and safety perceptions. Consequently, the observed treatment gaps in HFmrEF/HFpEF and in T2DM or CKD may partly reflect practice heterogeneity rather than systematic non-adherence to guidelines.

Fourth, the sample size, particularly within subgroups such as HFpEF, was modest, limiting statistical power and the precision of effect estimates. Observed transitions across HF phenotypes and phenotype-specific prescribing patterns should therefore be interpreted with caution.

Finally, this study did not systematically assess long-term clinical outcomes such as mortality, recurrent MI, HF hospitalization, or renal trajectory. As such, our results primarily describe prescription patterns, baseline characteristics, and predictors of SGLT2 inhibitor use, rather than providing direct evidence of therapeutic efficacy in the acute MI setting.

### 4.2. Clinical Implications and Future Directions

Future research should determine whether early initiation of SGLT2 inhibitors after acute MI provides incremental benefit beyond their established indications in HF, T2DM, and CKD. Ongoing and future randomized trials are expected to clarify whether systematic in-hospital initiation can improve ventricular remodeling, attenuate HF progression, or reduce long-term cardiovascular risk. Further research is particularly needed in HFpEF, HFmrEF, and non-diabetic post-MI populations, where therapeutic gaps remain substantial despite plausible mechanisms of benefit.

Developing standardized clinical pathways and refining risk stratification tools may help integrate SGLT2 inhibitors more consistently into post-MI care, ensuring that high-risk patients with HF, T2DM, or CKD are not overlooked. While robust evidence supports SGLT2 inhibitor use in these conditions, dedicated AMI trials have so far yielded neutral or mixed results on hard endpoints. Accordingly, current guidelines do not mandate early in-hospital initiation after AMI, but recommend SGLT2 inhibitors based on the presence of HF, diabetes, or CKD [43,44]. Our findings are consistent with this paradigm, showing that prescription was driven more by HF phenotype and CKD status than by AMI-related features alone, and they underscore the need for clearer guidance on the optimal timing and breadth of SGLT2 inhibitor use in the acute MI population.

Beyond their established glucose-lowering properties, SGLT2 inhibitors exert multiple pleiotropic effects that may be relevant in the post-AMI setting. Experimental and clinical data suggest favorable effects on myocardial energetics, inflammation, oxidative stress, autonomic regulation, and ventricular remodeling, independent of glycemic status [45,46,47].

Recent evidence has further highlighted potential electrophysiological and myocardial protective mechanisms that could be particularly relevant in patients with preserved or mildly reduced ejection fraction, supporting the biological plausibility of SGLT2 inhibitor use across the heart failure spectrum [26].

Taken together, these findings provide a rationale for future prospective, multicenter studies designed to evaluate the optimal timing, safety, and clinical impact of SGLT2 inhibitor initiation after AMI.

## 5. Conclusions

In this real-world cohort of patients with AMI who met guideline-based indications for SGLT2 inhibitors, prescription patterns were strongly influenced by HF phenotype and cardiometabolic comorbidities. SGLT2 inhibitors were preferentially initiated in patients with reduced LVEF, higher Killip class, and coexisting T2DM or CKD, while substantial therapeutic gaps persisted in HFpEF and in obese patients with metabolic or renal risk. Despite these disparities, early post-MI initiation was common and was not associated with major safety concerns.

Although SGLT2 inhibitors are not yet specifically recommended for routine use in the acute MI setting, our findings show that clinicians increasingly incorporate them into comprehensive post-MI management when established indications are present. The index hospitalization represents an important opportunity for early initiation, yet many eligible patients, especially those with HFpEF or overlapping metabolic risk, remain untreated.

Together, these observations highlight a selective but evolving adoption of SGLT2 inhibitors in post-MI care, shaped by HF severity and comorbidities. Future randomized trials and guideline updates are needed to clarify the role, timing, and breadth of SGLT2 inhibitor initiation in acute MI, particularly in non-diabetic and HFpEF populations, where potential benefits may be underutilized.

## Figures and Tables

**Table 1:** Baseline characteristics.

Variable	SGLT2 Inhibitor (+) (*n* = 117)	SGLT2 Inhibitor (−) (*n* = 63)	*p*-Value	SMD
**Demographics and Comorbidities**				
Age, years ± SD	60.85 ± 9.82	63.67 ± 11.58	0.046	0.26
Male sex, *n* (%)	95 (81%)	41 (65%)	0.016	0.36
Obesity ≥ 30 kg/m^2^, *n* (%)	34 (29%)	28 (44%)	0.038	0.32
Active smoker, *n* (%)	48 (41%)	21 (33%)	0.31	0.15
Hypertension, *n* (%)	96 (82%)	57 (90%)	0.13	0.24
HTN grade 3, *n* (%)	30 (26%)	26 (41%)	0.031	0.33
Diabetes mellitus, *n* (%)	51 (44%)	32 (51%)	0.4	0.14
CKD (eGFR ≤ 60 mL/min/1.73 m^2^), *n* (%)	15 (12.8%)	19 (30.15%)	0.004	0.43
HF NYHA class ≥ 2, *n* (%)	112 (95.7%)	62 (98.4%)	0.33	0.15
Prior MI, *n* (%)	18 (15%)	9 (14%)	0.8	0.03
**Admission Characteristics**				
SBP, mmHg (mean ± SD)	143.69 ± 26.03	145.16 ± 26.26	0.7	0.05
DBP, mmHg (mean ± SD)	87.26 ± 16.33	86.27 ± 16.34	0.5	0.06
Heart rate, bpm (mean ± SD)	81.09 ± 19.29	78.76 ± 17.86	0.3	0.12
**Type of ACS**				
STEMI, *n* (%)	103 (88%)	56 (89%)	0.86	0.02
Anterior wall MI, *n* (%)	56 (48%)	20 (32%)	0.03	0.33
Inferior wall MI, *n* (%)	43 (37%)	32 (51%)	0.06	0.28
NSTEMI, *n* (%)	14 (12%)	7 (11%)	0.86	0.02
Number of damaged vessels				
3, *n* (%)	40 (34%)	15 (24%)	0.14	0.23
2, *n* (%)	43 (37%)	25 (40%)	0.69	0.06
1, *n* (%)	34 (29%)	15 (24%)	0.45	0.11
Killip Classification ≥ II, *n* (%)	31 (26.5%)	8 (12.7%)	0.03	0.35
Hospitalization days, mean [IQR]	5.0 [3.0–7.0]	4.0 [3.0–7.0]	0.73	
**Laboratory Parameters**				
Troponin (ng/L), mean [IQR]	3592 [313.7–40,000]	1807 [121–34,383]	0.12	
Hemoglobin, g/dL, mean ± SD	14.62 ± 1.42	14.11 ± 1.60	0.053	0.10
Uric acid, mg/dL, mean ± SD	5.68 ± 1.8	5.82 ± 1.81	0.62	0.07
NLR, mean ± SD	6.22 ± 4.52	5.83 ± 6.22	0.06	0.07
PLR, mean ± SD	152.08 ± 77.16	150.22 ± 98.97	0.3	0.02
Na (mmol/L), mean ± SD	138.24 ± 2.98	138.54 ± 2.97	0.7	0.10
K (mmol/L), mean ± SD	4.06 ± 0.48	3.97 ± 0.52	0.3	0.18
Glycemia (mg/dL), mean ± SD	173.00 ± 76.85	165.54 ± 71.55	0.7	0.09
eGFR (mL/min/1.73 m^2^)	77.61 ± 20.10	73.46 ± 22.45	0.3	0.19
**Medication**				
ACEi/ARB/ARNI, *n* (%)	85 (73%)	43 (68%)	0.5	0.09
Beta-blocker, *n* (%)	91 (78%)	49 (78%)	0.9	0
MRA, *n* (%)	102 (87%)	48 (76%)	0.059	0.28
Loop diuretics	97 (83%)	49 (78%)	0.4	0.12
Statin, *n* (%)	116 (99.14%)	63 (100%)	0.46	0.13
Second antiplatelet, *n* (%)	117 (100%)	63 (100%)		
Ticagrelor, *n* (%)	66 (56%)	26 (41%)	0.052	0.30
Clopidogrel, *n* (%)	51 (44%)	37 (59%)	0.052	0.30
Anticoagulant, *n* (%)	29 (25%)	14 (22%)	0.700	0.06
**SGLT2 Inhibitor**				
Dapagliflozin, *n* (%)	88 (75%)	0%		
Empagliflozin, *n* (%)	29 (25%)	0%		
**Other Antidiabetic Drugs**	36	14	0.22	0.19
Metformin, *n* (%)	25 (21%)	8 (13%)		
Sulfonylurea, *n* (%)	9 (7.7%)	3 (4.8%)		
Insulin, *n* (%)	2 (1.7%)	3 (4.8%)		
**Echocardiography**				
LVEF (%), mean ± SD	38.21 ± 6.71	42.43 ± 8.34	0.001	0.57
HFpEF, *n* (%)	7 (6%)	20 (31.7%)		
HFmrEF, *n* (%)	51 (43.6%)	22 (34.9%)		
HFrEF, *n* (%)	59 (50.4%)	21 (33.3%)		
LVEDV (mL), mean ± SD	119.64 ± 45.59	106.81 ± 38.42	0.008	0.29
LVM, mean ± SD	236.26 ± 59.69	224.72 ± 64.28	0.094	0.18
LVEDD (cm), mean ± SD	5.00 ± 0.61	4.86 ± 0.60	0.2	0.23

**Table 2:** Evolution of HFrEF phenotype.

Evaluation	HF	SGLT2 Inhibitors	Control	*p*-Value
Baseline	HFrEF	59	21	
Follow-up	Remained HFrEF	30	6	0.078
Follow-up	Improved phenotype	29	15	0.078
Follow-up	HFmrEF	25	10	0.67
Follow-up	HFpEF	4	5	0.033

**Table 3:** Evolution of HFmrEF and HFpEF phenotypes.

Evaluation	HF	SGLT2 Inhibitors	Control	*p*-Value
Baseline	HFmrEF	51	22	<0.001
Follow-up	Remained HFmrEF	27	8	0.19
Follow-up	Became HFrEF	6	1	0.33
Follow-up	Improved to HFpEF	18	13	0.059
Baseline	HFpEF	7	20	<0.001
Follow-up	Remained HFpEF	4	16	0.23
Follow-up	HFmrEF	1	2	0.75
Follow-up	HFrEF	2	1	0.08

**Table 4:** Distribution of foundational and extended GDMT across HF phenotypes.

HF Phenotype	Total (*n*)	Foundational GDMT * *n* (%)	Extended GDMT ** *n* (%)
All HF	180	25 (13.8%)	68 (37.8%)
HFrEF	80	9 (11.3%)	36 (45.0%)
HFmrEF + HFpEF	100	16 (16.0%)	32 (32.0%)

* Foundational GDMT = ACEI/ARB/ARNI + β-blocker + MRA. ** Extended GDMT = Foundational GDMT + SGLT2 inhibitor.

**Table 5:** Model 1 of multivariable logistic regression—myocardial infarction severity.

Predictor	OR	95% CI	*p*-Value
Killip class	2.05	1.10–4.29	0.036
Male sex	2.08	0.98–4.44	0.056
Age	0.977	0.944–1.01	0.176
STEMI	0.718	0.223–1.95	0.488
Anterior MI	1.68	0.824–3.47	0.154
Hospital days	0.98	0.89–1.08	0.681
Number of damaged vessels	0.90	0.58–1.40	0.647

**Table 6:** Model 2 of multivariable logistic regression—comorbidities and risk factors.

Predictor	OR	95% CI	*p*-Value
Male sex	2.19	1.05–4.57	0.039
Obesity	0.43	0.21–0.87	0.019
Age	0.96	0.92–1.00	0.108
T2DM	0.93	0.43–2.00	0.656
eGFR	1.00	0.98–1.02	0.707
HTN	0.86	0.29–2.49	0.777
Prior MI	1.37	0.57–3.14	0.502
NLR	1.00	0.94–1.08	0.936

**Table 7:** Model 3 of multivariable logistic regression—medication and HF phenotype.

Predictor	OR	95% CI	*p*-Value
Age	0.966	0.933–0.999	0.048
Male sex	2.64	1.21–5.85	0.015
HFpEF	0.071	0.015–0.268	<0.001
HFmrEF	0.92	0.43–1.96	0.82
Killip	1.73	0.95–3.45	0.09
ACEi/ARB/ARNi	2.06	0.94–4.56	0.07
BB	0.802	0.334–1.85	0.61
MRA	0.42	0.106–1.36	0.17

**Table 8:** Model 4 of multivariable logistic regression—adjusted clinical model.

Predictor	OR	95% CI	*p*-Value
Age	0.96	0.92–1.00	0.078
Sex	2.58	1.14–5.93	0.023
HFpEF	0.081	0.016–0.317	0.007
HFmrEF	0.97	0.44–2.13	0.93
Killip	1.71	0.93–3.46	0.10
ACEi/ARB/ARNi	2.17	0.97–4.89	0.059
Obesity	0.61	0.29–1.32	0.20
STEMI	0.83	0.25–2.48	0.74
T2DM	1.02	0.48–2.15	0.96
eGFR	0.99	0.98–1.02	0.91
BB	0.85	0.34–2.05	0.718
MRA	0.43	0.11–1.43	0.193

## Data Availability

The data presented in this study are available on request from the corresponding author.

## Supplementary Materials

The following supporting information can be downloaded at: https://www.mdpi.com/article/10.3390/jcm15031056/s1, Table S1: Diagnostic performance of multivariable logistic regression models.
