# Supplementary material for: Patterns in Prescribing and Predictors of SGLT2 Inhibitor Administration in Patients with Heart Failure and Acute Myocardial Infarction: A Real-World Retrospective Cohort Study
Source: J Clin Med. 2026 Jan 28;15(3):1056. doi: 10.3390/jcm15031056 (PMC12898543; doi:10.3390/jcm15031056)
Supplement: Supplementary file 1 [file jcm-15-01056-s001.zip › jcm-4098841-supplementary.pdf]

Table S1. Diagnostic performance of multivariable logistic regression models

Model	AUC	Hosmer–Lemeshow p-value	Max VIF / GVIF^(1/(2Df))*
Model 1	0.68	0.47	1.20
Model 2	0.65	0.23	1.64
Model 3	0.76	0.83	1.34*
Model 4	0.76	0.71	1.36*

VIF = variance inflation factor; **GVIF***= generalized variance inflation factor adjusted for degrees of freedom; **AUC** = area under the ROC curve.
